# Prehospital response model and time to CT scan in blunt trauma patients; an exploratory analysis of data from the head injury retrieval trial

**DOI:** 10.1186/s13049-015-0107-1

**Published:** 2015-03-20

**Authors:** Alan A Garner, Kristy P Mann, Elwyn Poynter, Andrew Weatherall, Susan Dashey, Michael Puntis, Val Gebski

**Affiliations:** CareFlight, Locked Bag 2002 Wentworthville, Sydney, NSW 2145 Australia; NHMRC Clinical Trials Centre, University of Sydney, 92-94 Parramatta Road, Camperdown, Sydney, NSW 2050 Australia; Glenfield Hospital, Leicester, UK; Department of Anaesthesia, St George’s Hospital, London, UK

**Keywords:** Trauma, Prehospital, Physician, HEMS, Time, CT scan

## Abstract

**Background:**

It has been suggested that prehospital care teams that can provide advanced prehospital interventions may decrease the transit time through the ED to CT scan and subsequent surgery. This study is an exploratory analysis of data from the Head Injury Retrieval Trial (HIRT) examining the relationship between prehospital team type and time intervals during the prehospital and ED phases of management.

**Methods:**

Three prehospital care models were compared; road paramedics, and two physician staffed Helicopter Emergency Medical Services (HEMS) - HIRT HEMS and the Greater Sydney Area (GSA) HEMS. Data on prehospital and ED time intervals for patients who were randomised into the HIRT were extracted from the trial database. Additionally, data on interventions at the scene and in the ED, plus prehospital entrapment rate was also extracted. Subgroups of patients that were not trapped or who were intubated at the scene were also specifically examined.

**Results:**

A total of 3125 incidents were randomised in the trial yielding 505 cases with significant injury that were treated by road paramedics, 302 patients treated by the HIRT HEMS and 45 patients treated by GSA HEMS. The total time from emergency call to CT scan was non-significantly faster in the HIRT HEMS group compared with road paramedics (medians of 1.9 hours vs. 2.1 hours *P* = 0.43) but the rate of prehospital intubation was 41% higher in the HIRT HEMS group (46.4% vs. 5.3% *P* < 0.001). Most time intervals for the GSA HEMS were significantly longer with a regression analysis indicating that GSA HEMS scene times were 13 (95% CI, 7–18) minutes longer than the HIRT HEMS independent of injury severity, entrapment or interventions performed on scene.

**Conclusion:**

This study suggests that well-rehearsed and efficient interventions carried out on-scene, by a highly trained physician and paramedic team can allow earlier critical care treatment of severely injured patients without increasing the time elapsed between injury and hospital-based intervention. There is also indication that role specialisation improves time intervals in physician staffed HEMS which should be confirmed with purpose designed trials.

## Introduction

Traumatic brain injury (TBI) is one of the most significant causes of early trauma deaths and long-term morbidity, and is the leading cause of death in the first four decades of life in most developed countries. Apart from injury prevention programs little can be done to reduce the primary injury, but prompt control of subsequent secondary injury can be achieved by controlling factors such as hypoxia, hypotension and hypercapnia. A single prehospital observation of hypotension (systolic blood pressure < 90 mmHg) has been demonstrated to be one of the five strongest predictors of outcome and large observational data sets have found hypoxia in almost a quarter of patients which is significantly associated with increased morbidity and mortality [1]. The traditional trimodal distribution of trauma mortality is becoming out-dated and a greater proportion of deaths are now occurring in the immediate phase following injury [2,3]. This suggests that the timely delivery of advanced prehospital care may be critical in decreasing mortality and morbidity in modern trauma systems. Proponents of such interventions suggest that time providing appropriate treatment in the prehospital setting streamlines the subsequent delivery of care for the patient once they reach the emergency department (ED). For example, imaging or neurosurgical intervention would be more likely to occur without delay if interventions such as intubation had occurred prior to arrival at the ED. The optimal system to ensure patients receive these interventions as quickly as possible remains controversial.

The ideal prehospital system would allow early delivery of advanced level care while not delaying definitive care of the patient due to increased prehospital time. The purpose of this study is to assess the impact of a rapid-response helicopter emergency medical service (HEMS) system, deploying a physician and paramedic to the incident scene, on the resuscitation time in the ED and subsequent timing of imaging for TBI. There is also some evidence that differing organisational models and scope of operations in physician staffed HEMS (PS-HEMS) also produce different response times [4]. Data collected in the course of The Head Injury Retrieval Trial (HIRT) [5] allows a unique opportunity to explore the differences in prehospital and in-hospital time intervals for paramedic versus PS-HEMS systems, and also between two different PS-HEMS models.

## Methods

Following institutional ethics approvals the Head Injury Retrieval Trial (HIRT) (Clinicaltrials.gov NCT00112398) recruited patients in Sydney, Australia between May 2005 and March 2011. The trial was funded by Insurance Australia Group and the NSW Motor Accidents Authority. The HIRT system was able to rapidly identify trauma patients using web based access to the NSW Ambulance (NSWA) Computer Assisted Dispatch System (CAD). Patients were then randomised via a central computerised randomisation process, to either a standard paramedic Emergency Medical Service (EMS) response or the dispatch of a physician and paramedic team by helicopter; the ‘HIRT HEMS’ response. Details of both the trial methodology [5] and the case identification and dispatch system [6] have been reported elsewhere.

A physician response model existed within the recruiting catchment area prior to commencement of the trial. This system was tasked by the NSWA dispatch system to prehospital trauma cases by road or helicopter. However, historical data indicated that physician response was activated for less than 5% of severe head injuries. In December 2007, two and a half years after commencement of recruitment in HIRT, a proactive tasking system which mimicked components of the trial case identification system was implemented by NSWA to identify severe trauma cases for physician response referred to as the Rapid Launch Trauma Coordinator (RLTC). The adult case identification system for HIRT and the RLTC operated independently without knowledge of the others actions until physician teams were activated and had departed the operations bases. In many cases both systems responded a physician team to the same patient in parallel, producing a unique opportunity to directly compare the physician response models. The HIRT and RLTC dispatch systems worked collaboratively to identify paediatric cases, as age less than 16 years was an exclusion criteria from the trial. A comparison of the two systems in identifying severely injured children and the effect on the paediatric trauma system in Sydney has been previously reported [6].

In all cases identified as possibly having a significant head injury by the HIRT case identification system and subsequently enrolled in the trial, a paramedic response was sent by road ambulance. Cases randomised to the treatment group were also attended by the HIRT HEMS. Cases independently identified as requiring a physician level response by the RLTC had a NSWA physician team dispatched. The RLTC activated NSWA physician teams to patients in both the control and treatment groups of the trial as they were unaware of treatment allocation by the trial randomisation system. There were therefore 4 dispatch scenarios:

1. Road ambulance paramedic system only (allocated to standard care by the trial randomisation system and not identified by the RLTC).
2. HIRT HEMS response in addition to road paramedics (allocated to physician treatment by the trial randomisation system and not identified by the RLTC).
3. NSWA physician response in addition to road paramedics (allocated to standard care by the trial randomisation system and identified by the RLTC).
4. Both NSWA physician and HIRT HEMS responses in addition to road paramedics (allocated to physician treatment by the randomisation system and also identified by the RLTC).

**Figure 1:**
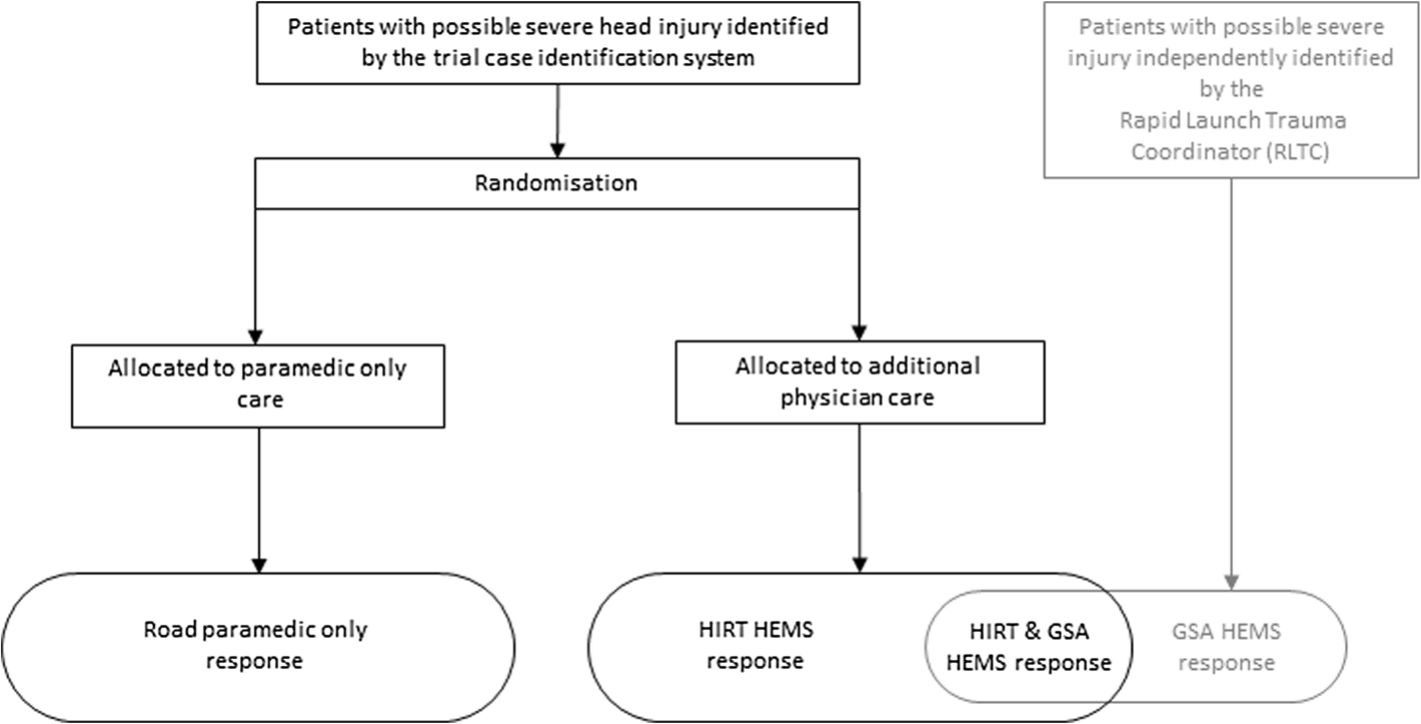
**Case identification and dispatch systems utilised during the HIRT.** The trial case identification system identified patients with possible severe head injury and randomised them to either paramedic only care or additional physician care. The Rapid Launch Trauma Coordinator (RLTC) independently identified some of the same patients and dispatched the GSA HEMS physician team. Therefore teams dispatched to patients consisted of paramedics alone, or in combination with HIRT HEMS, GSA HEMS or both physician services.

The dispatch systems are depicted in Figure 1.

When both medical teams responded to a case, the first team to arrive on scene assumed responsibility for the patient. The other team was usually stood down by the central coordination system, although at times both teams arrived on scene particularly where there were multiple patients. The physician bases are seven nautical miles (nm) apart, both located in western Sydney. The HIRT HEMS responded to all cases by helicopter regardless of distance, and all physicians held specialist qualifications in Emergency Medicine, Anaesthesia or Intensive Care. The NSWA physician teams responded by road ambulance or helicopter depending on the distance from their operations base. NSWA physicians were either specialists of the same background as the HIRT physicians or advanced trainees.

Standard care by paramedics only was according to written protocols of the NSWA and included: cannulation and up to 1 Litre intravenous crystalloid infusion; ventilation via supraglottic airways and bag-valve-mask ventilation; intubation without neuromuscular blockade; needle chest decompression; midazolam for seizures or sedation; analgesia with methoxyflurane and morphine; and splinting and spinal immobilization. Interventions additional to standard care by either of the physician teams included: anaesthesia with neuromuscular blockade and induction agents such as Thiopentone or Ketamine; surgical airways; needle cricothyroidotomy; tube or open thoracostomy; administration of 7.5% saline or 20% mannitol; and administration of packed red blood cells. All patients were transported to the nearest Level 1 trauma centre. After arrival in the trauma centre all care was directed by the standard policies of that institution including procedures in the ED and transfer to CT scan.

Patients are reported who met the definition of severe injury used in the Head Injury Retrieval Trial [5] which included any of the following:

- Died at any time prior to hospital discharge
- Admission to High Dependency Unit (HDU) or Intensive Care Unit (ICU)
- Four or more rib fractures
- Insertion of tube thoracostomy/s
- Spinal cord injury with deficit
- Blood transfusion > 4 units packed red blood cells in the first 24 hours
- Required laparotomy, thoracotomy, craniotomy or interventional radiology
- One or more fractured femurs or fractured pelvis requiring fixation / embolization
- Burns > 20% Body Surface Area or intubation for airway burns
- Fall in GCS of 2 or more points from initial GCS (if not drug induced) in first 4 hours post arrival in ED
- Documented to be in Post Traumatic Amnesia for more than 1 week post injury

Times data were collected from the NSWA CAD system, and the case sheets of the responding prehospital units. Time of departure from the ED was obtained from the patient’s hospital medical record, and the time of commencement of the CT scan was obtained from the CT scan film/file. Whether the patient was trapped at the scene and interventions both prehospital and in the receiving hospital ED which may have affected time intervals were abstracted from the prehospital treatment records of the responding teams and the patient’s hospital medical record as per the Human Research Ethics Approval for the HIRT. Seniority of the physician (specialist or trainee) in the two PS-HEMS models was also noted.

Time point definitions used are:

- First key stroke (FKS): time of commencement of the emergency call when the first data item was entered into the CAD system by the call taker.
- Depart base: response unit leaves their operations base
- Patient contact: responding team makes physical contact with the patient
- Depart scene: patient began to move from the incident scene
- Arrive ED: patient arrived in the bed space of the receiving ED
- CT: time recorded on CT scan film/file
- NSWA team notified: RLTC contacts NSWA team

Time intervals investigated therefore are:

- Tasking & mobilisation time: FKS to depart Base
- Response time: depart base to patient contact
- Scene time: patient contact to depart scene
- Transport time: depart scene to arrive ED
- Total prehospital time: FKS to arrive ED
- ED time: arrive ED to CT
- Time to diagnosis: FKS to CT

The time from FKS to NSWA physician team notified (tasking time) and the time from this notification for the team to depart base (mobilisation time) are also reported as separate items. These discrete time intervals were not available for the HIRT HEMS as the case identification and mobilisation processes partly overlapped.

The response model of the NSWA physician teams evolved over the time the study was recruiting. In May 2007 two previous services were combined into a single service operating from a new base with a new helicopter contractor which became known as the Greater Sydney Area HEMS (GSA HEMS). The RLTC was introduced in December 2007. Data for the NSWA physician response model are reported only after December 2007 when the model had reached its final configuration, the GSA HEMS.

### Statistical analysis

This study is an exploratory analysis of time intervals according to the actual treatment received and conforms to the STROBE Statement [7] on reporting of observational studies. Prehospital time intervals according to intention-to-treat analysis have been reported with the main trial outcomes [8]. Baseline characteristics and outcome data were compared between the three groups by one of three tests. An Analysis of Variance (ANOVA) was used to test for differences for normally distributed data, and this data was summarised with mean (standard deviation (SD)). When data did not follow a normal distribution, median and interquartile ranges were used to summarise the data and a Kruskal Wallis test was used for testing for differences. Chi squared tests were used for categorical data, except in cases of low cell counts when a Fishers exact test was used. A multivariate regression model was developed for time spent on scene by the physician teams using backwards selection of physiological variables, interventions performed on scene and entrapment status. Only variables identified as p < 0.20 on univariate analysis were considered for inclusion in the regression. No adjustments have been made for multiple comparisons and p < 0.05 was considered statistically significant. All analysis was undertaken in SAS v9.3 (Cary, USA).

## Results

**Figure 2:**
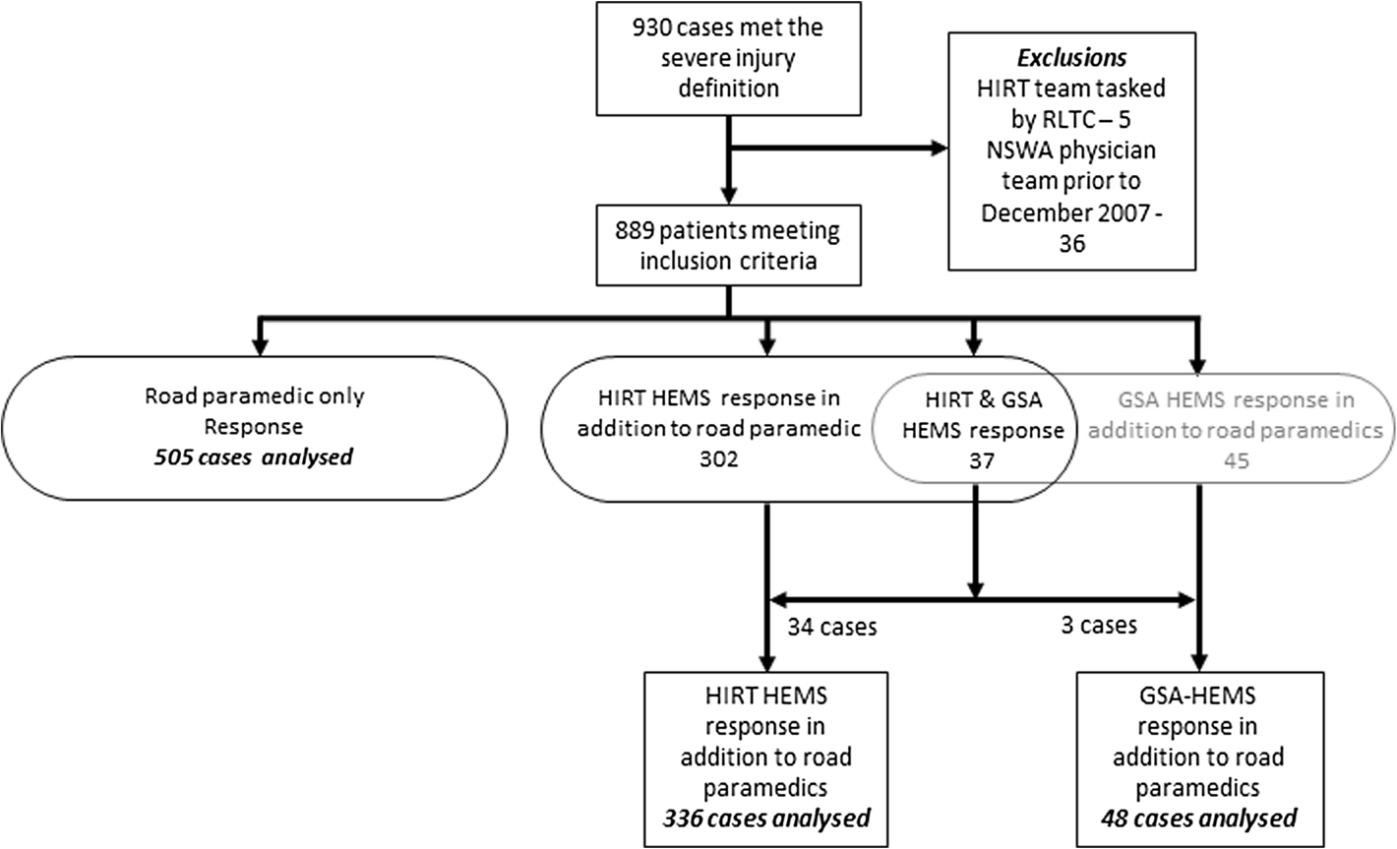
**Flowchart of patients meeting the trial severe injury criteria into treatment groups showing analysis exclusions.**

Over the nearly six-year period of trial recruitment between May 2005 and March 2011, 3125 incidents were randomised, yielding 930 patients who met the inclusion criteria for significant injury as per the trial protocol [5]. Thirty six of the 930 patients were excluded as they were treated by NSWA physician teams prior to December 2007 and 5 patients were excluded as they were patients allocated to standard care but treated by the HIRT medical team at the direction of the RLTC when no GSA HEMS team was available. This resulted in 889 patients included in the final analysis. Numbers of patients included in each group are detailed in Figure 2.

In 37 cases both a HIRT and GSA HEMS team were dispatched in parallel to the same case. In 22 of these cases the GSA HEMS Sydney base was closer to the incident location than the HIRT HEMS base, however the GSA HEMS arrived first at the incident location in 3 cases (P < 0.001). Two of the three cases were near the limit of operational range of the HIRT HEMS and were accessed by physician teams from rural GSA HEMS bases, and in the third case GSA HEMS responded by road 1 Nm from their Sydney base.

The baseline characteristics of the patients for the three response models are shown in Table 1. Patients identified by the RLTC and treated by GSA HEMS were younger and more likely to have transportation trauma injuries than patients treated by either road paramedics or the HIRT HEMS. Patients treated by the HIRT HEMS however had significantly higher New Injury Severity Scale (NISS) scores and lower Glasgow Coma Scale (GCS) scores.

**Table 1:** **Patient characteristics by treatment group**

	**Road paramedic (n = 505)**	**HIRT HEMS (n = 336)**	**GSA HEMS (n = 48)**	**p-value**
**Patient age (years)**	46.3 (20.5)	45.7 (21.4)	37.3 (18.2)	0.02,^§^^¥^
**Gender (male)**	360 (71%)	252 (75%)	34 (71%)	0.5
**Mechanism of injury**				
**- Transport**	318 (63%)	219 (65%	44 (92%)	0.003,^§^^¥^
**- Fall**	137 (27%)	86 (26%)	3 (6%)	
**- Other**	50 (10%)	31 (9%)	1 (2%)	
**ISS**	21.3 (13.3)	23.2 (15.1)	21.7 (11.4)	0.13
**NISS**	27.2 (16.9)	30.2 (18.1)	27.1 (15.1)	0.04,*
**First GCS**	13 (8–15)	10 (4–14)	13 (6–15)	<0.001,*^¥^
**First SBP (mmHg)**	113.5 (37.8)	114.3 (41.9)	103.3 (43.1)	0.20

Statistics reported as N (%), mean (SD) or median (IQR) as appropriate. ISS, Injury Severity Scale. NISS, New Injury Severity Scale. GCS, Glasgow Coma Scale. SBP, Systolic blood pressure.

*indicates difference between Road Paramedics and HIRT HEMS at <0.05 level.

^§^indicates difference between Road paramedics and GSA HEMS at <0.05 level.

^¥^indicates difference between HIRT HEMS and GSA HEMS at <0.05 level.

Interventions by team type are detailed in Table 2 for both the prehospital and ED phases of care. The proportion of patients receiving interventions in each phase was similar between the two physician groups, with the exception of thoracic decompression and pelvic circumferential compression device application. There were however significant differences in prehospital cannulation rates, intubation rates, and administered fluid volumes between the two physician groups and the paramedic treatment group. The rate of intubation in the ED for road paramedic treated patients was also significantly higher than the rate in either of the physician teams.

**Table 2:** **Intervention rates prehospital and in the ED by treating team model**

	**Road paramedics (n = 505)**	**HIRT HEMS (n = 336)**	**GSA HEMS (n = 48)**	**p-value**
**Peripheral cannulation**				
*Prehospital*				
- 0	122 (24%)	16 (5%)	1 (2%)	<0.001,*^§^
- 1	375 (74%)	222 (66%)	34 (71%)	
- 2 or more	8 (2%)	98 (29%)	13 (27%)	
*Emergency department*				
- 0	99 (20%)	88 (27%)	10 (21%)	0.11
- 1	255 (51%)	161 (49%)	28 (60%)	
- 2 or more	144 (29%)	81 (25%)	9 (19%)	
**Intubation**				
*Prehospital*	27 (5%)	156 (46%)	20 (42%)	<0.001,*^§^
*Emergency department*	164 (33%)	37 (11%)	3 (6%)	<0.001,*^§^
**Thoracic decompression**				
*Prehospital*	2 (0.4%)	4 (1.2%)	6 (12.5%)	<0.001,^§^^¥^
*Emergency department*	54 (11%)	39 (12%)	8 (17%)	0.45
**Administration of intravenous fluids (mls)**				
*Prehospital*	0 (0–200)	500 (100–1000)	725 (200–1375)	<0.001,*^§^^¥^
*Emergency department*	1000 (500–2000)	1000 (500–1800)	1000 (500–1500)	0.32
**Administration of prehospital packed red blood cells**				
*Any amount*	0	20 (6%)	4 (8%)	<0.001,*^§^
**Application of femur traction splint**				
*Prehospital*	1 (0.2%)	4 (1.2%)	1 (2%)	0.06
*Emergency department*	15 (3%)	14 (4%)	3 (6%)	0.29
**Application of prehospital pelvic compression device**	1 (0.2%)	11 (3%)	6 (13%)	<0.001,*^§^^¥^

Statistics reported as N (%), mean (SD) or median (IQR) as appropriate.

*indicates difference between Road Paramedics and HIRT HEMS at <0.05 level.

^§^indicates difference between Road paramedics and GSA HEMS at <0.05 level.

^¥^indicates difference between HIRT HEMS and GSA HEMS at <0.05 level.

Table 3 details the time intervals for each phase of care by prehospital team model.

**Table 3:** **Time intervals for each phase of care by prehospital treatment model**

	**Road paramedics (n = 505)**	**HIRT HEMS (n = 336)**	**GSA HEMS (n = 48)**	**p-value**
**Time to team arrival at patient from FKS** (mins)	10 (7–14)	18 (16–21)	39 (33–48)	<0.001,*^§^^¥^
**Total scene time** (mins)	17 (12–26)	25 (18–35)	56 (43–69)	<0.001,*^§^^¥^
**Transport time** (mins)	16 (10–23)	15 (11–20)	17 (13–25)	0.04,^§^^¥^
**Total prehospital time** (mins)	46 (34–58)	53 (41–58)	92 (79–116)	<0.001,*^§^^¥^
**Time from ED arrival to CT scan** (mins)	70 (45–116)	59 (39–95)	74 (49–106)	<0.001,*^§^^¥^
**Time from FKS to CT scan** (hours)	2.0 (1.5-2.9)	1.9 (1.6-2.6)	3.0 (2.3-3.8)	<0.001,^§^^¥^

Statistics reported as median (IQR). FKS, First Key Stroke of emergency call into computerised dispatch system. ED, Emergency Department. CT, Computerised Tomography.

*indicates difference between Road Paramedics and HIRT HEMS at <0.05 level.

^§^indicates difference between Road paramedics and GSA HEMS at <0.05 level.

^¥^indicates difference between HIRT HEMS and GSA HEMS at <0.05 level.

### Subgroup comparison of patients receiving physician care

The median time from FKS to notification of the GSA HEMS by the RLTC (tasking time) was 8 minutes (IQR 4–13). The median time from notification to departing the base (mobilisation time) for the GSA HEMS was 8 minutes (IQR 5–11). This varied by vehicle being 5 minutes (IQR 2–6) for road vehicles and 10 minutes (IQR 7–12) for helicopter responses. In two GSA HEMS helicopter cases the mobilisation time was 0 minutes as they were diverted whilst airborne. This was not possible with HIRT HEMS as the CAD system was available to them only at their operations base.

Table 4 details the rate of patient entrapment for the two physician teams from December 2007.

**Table 4:** **Entrapment rates in the two physician teams**

	**Never trapped**	**Trapped, extricated prior to physician team arrival**	**Trapped, extricated after physician team arrival**
GSA HEMS n = 46	19 (41%)	13 (28%)	14 (30%)
HIRT HEMS n = 165	105 (64%)	9 (5%)	51 (31%)

Data for cases from December 2007 to March 2011 (see Methods).

Significantly more patients were trapped at the time of dispatch of GSA HEMS (P < 0.001). However many of these patients had been extricated by the time of GSA HEMS arrival and the rates that remained trapped when either of the physician teams made patient contact were similar.

Table 5 compares the time intervals between the two physician teams. All time intervals were significantly shorter for the HIRT HEMS including for the subgroups of patients who were not trapped at the time of physician team contact, and for the same subgroup but considering intubated patients only.

**Table 5:** **Time intervals for physician treated patients after December 2007**

	**HIRT HEMS**	**GSA HEMS**	***P*** **value**
**Combined tasking & mobilisation time (mins)**	7 (6–8), *n = 336*	17 (13–24), *n = 46*	<0.001
**Physician team scene time (mins) for all patients treated**	18 (10–28), *n = 328*	32 (25–46), *n = 43*	<0.001
*Subgroup of patients not trapped at the time of physician team contact with the patient*			
**Physician team scene time (mins)**	13 (9–21), *n = 110*	31 (23–46), *n = 30*	<0.001
**Physician team scene time (mins) for patients requiring prehospital intubation**	16 (10–23), *n = 54*	34 (25–51), *n = 16*	<0.001

All values reported as medians (IQR). “Physician team scene time for all patients treated” is less than the “Duration total scene time” in Table 3 as a road ambulance team was usually the first to arrive on the scene with the physician team arriving subsequently.

Further analysis of the GSA HEMS data by doctor grade and vehicle type was conducted seeking possible explanations for the differences seen in Table 5. For the GSA HEMS median scene time for the specialists was 35 minutes (IQR: 26–46) compared with the registrars which was 32 minutes (IQR 25–46), *P = 0.72.*

Table 6 details the distance to the scene and response time by physician team and vehicle. When the response time was corrected for the distance from the base to the incident scene, GSA HEMS response by road was significantly slower than response by either helicopter (*P* <0.001).

**Table 6:** **Distance to the scene and response time by physician team and vehicle**

	**GSA HEMS by road, n = 11**	**GSA HEMS by helicopter, n = 37**	**HIRT HEMS, all by helicopter, n = 335**	***P***
Nm from base to incident	5 (2–7)	20 (14–28)	11 (7–16)	<0.001,*^§^^¥^
Response time, minutes	15 (10–19)	22 (19–27)	11 (9–14)	<0.001,*^¥^

Nm, nautical mile. All values reported as medians (IQR).

*indicates difference between GSA HEMS by road and GSA HEMS by helicopter at <0.05 level.

^§^indicates difference between GSA HEMS by road and HIRT HEMS at <0.05 level.

^¥^indicates difference between GSA HEMS by helicopter and HIRT HEMS at <0.05 level.

A regression analysis was conducted utilising the physiological variables, interventions performed on scene and entrapment status as inputs to further explore the relationship between scene times in the two physician groups. Factors independently associated with scene time are detailed in Table 7.

**Table 7:** **Results of multiple regression analysis showing factors independently associated with longer scene times in physician treated patients**

**Parameter**	**Mean additional scene time in minutes (95% CI)**	**P value**
Pelvic compression device (compared to none)	11 (3–19)	0.008
Thoracic decompression (compared to none)	24 (11–36)	<0.001
GSA HEMS treatment (compared to HIRT HEMS)	13 (7–18)	<0.001
Entrapment when physician team present (compared to no entrapment when physician team present)	19 (14–23)	<0.001

## Discussion

An optimally designed trauma system requires a patient-centred approach to care. The patient needs a prehospital response that delivers them the most appropriate level of care as quickly as possible after their injury with rapid case identification, tasking of the clinical team with the most appropriate skills that will reach the patient most quickly and a clear focus on both undertaking time critical interventions and moving the patient quickly through their treatment path. The HIRT system appears to display advantages in all these areas.

Patients treated by the HIRT HEMS model had significantly longer total prehospital times than patients treated by paramedics, but significantly shorter times from ED arrival to CT scan. The overall effect was non-significantly shorter times from injury to CT scan in the HIRT HEMS group. The longer prehospital time in the HIRT HEMS group was however associated with a 41% greater prehospital intubation rate. This suggests that well-rehearsed and efficient interventions carried out by a highly trained physician and paramedic team enables earlier critical care treatment of severely injured patients during the prehospital phase. Importantly this can be achieved without increasing the time between injury and definitive diagnosis via CT scan which allows planning for urgent surgical intervention if required.

It has been postulated that medical intervention by doctors on scene results in delay to hospital admission and hence delay to definitive care. Definitions of the point at which definitive care is achieved have varied in previous studies [9]. For patients with a head injury not necessitating surgical drainage of a haematoma, control of secondary factors that may exacerbate the primary injury constitutes definitive care. Advance intervention teams can often achieve this in the prehospital phase. For those that do require surgical intervention time of CT indicates the point at which the head injury patient has been resuscitated and imaged, enabling planning for surgery. A previous retrospective analysis also suggested that despite longer scene times the total resuscitation time is not prolonged when physicians perform prehospital intubation [10] and that prehospital interventions reduce the primary survey time in the emergency department [11]. In our regression analysis prehospital intubation by a physician team was also not independently associated with longer scene times. Time spent in the emergency department prior to CT varied with physician model however.

As the NSWA paramedic protocols exclude the use of sedative and neuromuscular blocking drugs the difference in intubation rates between the paramedic team and both physician teams is an expected finding. However the time intervals for the GSA HEMS were significantly longer in almost every interval examined when compared with both the paramedic and HIRT HEMS models and GSA HEMS treatment was associated with a 13 minute longer scene time independent of injury severity, interventions performed or entrapment when compared with HIRT HEMS in a regression model. The difference between the physician teams was not expected as both the skills of the physicians and the rates of intubation (the most common major intervention) were similar. A recent study [12] comparing physician staffed HEMS in Germany and the Netherlands found significantly different overall prehospital times between physician staffed HEMS models in the two countries and counter intuitively the faster service had the higher prehospital intervention rate. Many complex differences in prehospital strategy between the countries were demonstrated but the implications remained unclear. Our study however has the advantage of being performed in a single trauma system with the physician teams being dispatched in parallel to the same patient in many cases.

We attempted to remove the potential confounding effects of entrapment and differing GCS scores by analysing subgroups of non-trapped and intubated patients and by conducting a regression analysis but large differences in scene time between physician groups were still observed. The seniority of the physicians in the GSA HEMS also did not appear to explain the observed difference as times were similar for specialists and registrars. It is possible that differences in policies and/or processes resulted in the observed differences but further studies would be required to examine this.

Time from ED arrival to CT scan for the HIRT HEMS patients is significantly faster than both the paramedic and GSA HEMS groups. That the HIRT HEMS was faster than the paramedic group is probably due to the lower number of interventions required in the ED. Again however the difference between physician models is unexpected as the ED intervention rates are similar and after ED arrival all patients are treated under the policies and procedures of the receiving trauma centre rather than the prehospital team. Differences in handover practices could perhaps have caused the observed difference but again this finding requires further investigation.

There are significant differences in the scope of operations between the HIRT and GSA HEMS teams. The HIRT HEMS only conducted prehospital responses, responses that did not require hoisting operations, and in an area less than 60 nm of their base. The GSA HEMS however conduct both interfacility and prehospital transports as well as hoisting operations from anywhere within NSW. There is evidence from European studies that mobilisation time for mountain rescue missions is shorter in dedicated HEMS services when compared with operations that include military and police duties [4]. Although the GSA HEMS is dedicated to HEMS operations it is likely that the degree of specialisation of the HIRT HEMS model simplified procedures resulting in shorter mobilisation times. The operator that provided both the HIRT helicopter and physician team (CareFlight) previously provided a multirole HEMS operation in Sydney with mobilisation times that were similar to those reported here for the GSA HEMS teams. This combined with the previous European data suggest that role specialisation is an important factor in lowering mobilisation times. Other operational contributing factors may have been:

- Base location: the GSA HEMS operations base is located at a busy general aviation airport whereas the HIRT HEMS base is stand alone and outside of controlled air space. This may partially explain the differences in observed mobilisation times for responses by helicopter.
- Road responses: GSA HEMS responses by road vehicle showed significantly longer transit times to the scene compared with helicopter responses when the distance from the base was controlled for. Sydney is a geographically large urban area with problematic road transportation infrastructure. Rapid response by helicopter via the HIRT HEMS model appears to produce faster access times than road response in such an environment, even over short distances. This is supported by the arrival of the HIRT HEMS at the patient prior to the Sydney based GSA HEMS team in all but one occasion of parallel tasking, despite the GSA HEMS responding over short distances by road to locations to which they were closer than the HIRT HEMS team.

The pool of physicians and paramedics is much larger in the GSA-HEMS system compared with HIRT HEMS. GSA HEMS had more than 45 physicians and 20 paramedics resulting in over 900 possible physician-paramedic pairs, compared with 12 physicians and 2 paramedics with 24 possible pairs in HIRT HEMS. Small clinical staff pool sizes also characterise many European HEMS such as in Norway [13]. High levels of team familiarity have been associated with significantly faster theatre turn-around times in orthopaedic surgery [14] and lower surgical and teamwork errors in cardiac surgery [15]. A possible correlation between prehospital team member familiarity and scene times would require further exploration. Whether the specialisation of the HIRT HEMS contributed to shorter scene times and ED arrival to CT scan transit times would also require evaluation with further studies.

The HIRT and RLTC dispatch systems have been compared previously regarding accuracy in identifying severely injured children and the subsequent effect on the prehospital paediatric trauma system in Sydney [6]. In addition to being more accurate, the current study also suggests that the HIRT case identification system is faster than the RLTC with HIRT HEMS having identified the case and become airborne in a shorter time than it took the RLTC to notify GSA HEMS. This difference in dispatch systems was a major contributor to the observed difference between the two HEMS systems in the time to depart the base from FKS.

At cessation of randomisation into the HIRT in March 2011 NSWA withdrew access by the HIRT HEMS team to the CAD screens. Tasking of both HEMS since that time has been exclusively by the RTLC utilising dispatch policies introduced by NSWA without consultation with HIRT HEMS. The current NSWA tasking policy directs that the closest available physician team should be dispatched regardless of differences in operational capability between the services. Additionally a motorway that runs between the HEMS bases is used as a guide by the RLTC to allocate cases. The motorway is 1.5 nm from the HIRT HEMS base and 5.5 nm from the GSA HEMS base. This study indicates that review of both the case identification process and team allocation is warranted.

### Limitations

The most obvious limitation of this study is that it is an exploratory analysis of data from a randomised controlled trial that was designed to answer a different question. There is evidence for example, of case selection in the patients identified by the RLTC for GSA HEMS response who were younger, and more likely to be a trapped victim of a transportation incident than patients who were treated by either road paramedics or HIRT HEMS. Patients treated by HIRT HEMS however were more severely injured as measured by the NISS and GCS scores. Attempts were made to control for these factors by analysing subgroups which removed some of the confounding variables such as entrapment and requirement for intubation, and by performing a regression analysis.

Despite these limitations the cases of parallel tasking of the two physician response models to the same cases provides a direct comparison of the total time to patient access for the two end-to-end case identification, mobilisation and response systems, with the proviso that the distance from the respective operations base to the scene is controlled for. The fact that in most parallel tasking cases the GSA HEMS was closer but did not arrive first at the patient suggests that the observed differences are not due to selection as both services were responding to the same patient simultaneously.

The estimates for the additional scene time required to perform thoracic decompression or apply a pelvic compression device by physician teams are unexpectedly long. Absolute numbers of patients requiring these interventions were very small however resulting in wide confidence intervals. A recent study from the German national trauma registry demonstrated an additional 3.2 mins was required on scene by physician teams to establish a chest tube in a sample of more than eleven hundred patients requiring the procedure [16].

As shown by the HIRT study [5], physician staffed retrieval teams are now an established component of standard care in the Sydney prehospital system. The opportunity to answer the key hypothesis posed by the HIRT study in NSW has therefore been lost and attention should now focus on the best means to deliver physician care to critically injured patients. This study suggests that case identification systems in Sydney need to be reviewed, and that times for response and treatment between different physician response models may not be equivalent and should be taken into account during tasking decisions.

## Conclusion

This study suggests that well- rehearsed and efficient interventions carried out on-scene, by a highly trained physician and paramedic team can allow earlier critical care treatment of severely injured patients without increasing the time elapsed between injury and hospital-based intervention. There is also an indication that role specialisation improves time intervals in PS-HEMS which should be confirmed with purpose designed trials.

## Abbreviations

CAD: Computer Aided Dispatch
CT: Computerised Tomography
ED: Emergency Department
EMS: Emergency Medical Service
FKS: First Key Stroke
GCS: Glasgow Coma Score
GSA: Greater Sydney Area
HEMS: Helicopter Emergency Medical Service
HIRT: Head Injury Retrieval Trial
ISS: Injury Severity Score
IQR: Interquartile Range
NISS: New Injury Severity Score
Nm: Nautical mile
NSWA: New South Wales Ambulance
NSW: New South Wales
PS-HEMS: Physician Staffed Helicopter Emergency Medical Service
RLTC: Rapid Launch Trauma Coordinator
SBP: Systolic Blood Pressure
TBI: Traumatic Brain Injury
